# Journal of Neurodevelopmental Disorders reviewer acknowledgement 2012

**DOI:** 10.1186/1866-1955-5-5

**Published:** 2013-03-21

**Authors:** Joseph Piven

**Affiliations:** 1 Carolina Institute for Developmental Disabilities, University of North Carolina at Chapel Hill, 100 Renee Lynne Court, Carrboro, NC, 27510, USA

## Abstract

**Contributing reviewers:**

The editors of *Journal of Neurodevelopmental Disorders* would like to thank all of our reviewers who have contributed to the journal in volume 4 (2012). High quality and timely reviews are critical to the overall quality of the journal. We are committed to providing a unique and important outlet for scholarship regarding neurodevelopmental disorders and are indebted to the outstanding reviewers who have contributed their time over the last year in helping us to reach this goal.

## 

Melissa Allen

United Kingdom

Elizabeth Aylward

United States of America

Margaret Bauman

United States of America

Raphael Bernier

United States of America

Elina Birmingham

Canada

Dorothy Bishop

United Kingdom

Susan Bookheimer

United States of America

Marc Brysbaert

Belgium

Linda Campbell

Australia

Neva Corrigan

United States of America

Ana Cubillo

United Kingdom

Geraldine Dawson

United States of America

Saumitra Deb

United Kingdom

**Manny DiCicco**-**Bloom**

United States of America

Maria Luz Dizon

United States of America

Jed Elison

United States of America

Deborah Fidler

United States of America

Carsten Finke

Germany

Chris Frith

United Kingdom

Susanna Fryer

United States of America

Dennis Grayson

United States of America

Paul Hagerman

United States of America

Francesca Happe

United Kingdom

Todd Hare

Switzerland

Joost Janssen

Netherlands

Warren Jones

United States of America

Emily Jones

United States of America

**Annette Karmiloff**-**Smith**

United Kingdom

Cary Kogan

Canada

Gregor Kohls

United States of America

**Meng**-**Chuan Lai**

United Kingdom

Amir Levine

United States of America

Alice Lin

United States of America

Rhiannon Luyster

United States of America

Carla Mazefsky

United States of America

James McPartland

United States of America

Nancy Minshew

United States of America

Sandra Mooney

United States of America

Jeff Munson

United States of America

Charles Nelson

United States of America

Joshua New

United States of America

Richard Nowakowski

United States of America

**John O**'**Doherty**

United States of America

Noriko Osumi

Japan

Daniela Plesa Skwerer

United States of America

Armin Raznahan

United States of America

Allan Reiss

United States of America

Lawrence Reynolds

United States of America

Todd Richards

United States of America

Cary Savage

United States of America

Gaia Scerif

United Kingdom

Stephen Scherer

Canada

Emily Simonoff

United Kingdom

Mikle South

United States of America

Catherine Stoodley

United States of America

Bruce Tomblin

United States of America

Anna Tury

United States of America

Jaap van Pelt

Netherlands

Katarina Varnäs

Sweden

David Walker

Australia

Karli Watson

United States of America

Zhengang Yang

China

